# Collaborative advance care planning in advanced cancer patients: col-ACP –study – study protocol of a randomised controlled trial

**DOI:** 10.1186/s12904-020-00629-7

**Published:** 2020-08-24

**Authors:** Carola Seifart, Martin Koch, Nico Leppin, Katharina Nagelschmidt, Jorge Riera Knorrenschild, Nina Timmesfeld, Winfried Rief, Pia von Blanckenburg

**Affiliations:** 1 Institutional Review Board, Clinical Ethics, Philipps-University Marburg, Marburg, Germany; 2 Department of Clinical Psychology and Psychotherapy, Philipps-University Marburg, Gutenbergstraße 18, 35032 Marburg, Germany; 3 Department of Internal Medicine, Division Hematology and Oncology, University Clinic of Gießen and Marburg, Marburg, Germany; 4 Department of Medical Informatics, Biometry and Epidemiology, Ruhr-University Bochum, Bochum, Germany

**Keywords:** Advance care planning, Psycho-oncological intervention, Palliative care, End of life care, Complex intervention

## Abstract

**Background:**

To assure patient-centred end-of-life care, palliative interventions need to account for patients’ preferences. Advance care planning (ACP) is a structured approach that allows patients, relatives and physicians to discuss end-of-life decisions. Although ACP can improve several patient related outcomes, the implementation of ACP remains difficult. The col-ACP-study (collaborative advance care planning) will investigate a new ACP procedure (col-ACP-intervention (German: Hand-in-Hand Intervention)) in palliative cancer patients and their relatives that addresses individual values and targets barriers of communication before an ACP process.

**Methods:**

In a randomised controlled trial, 270 cancer patients without curative treatment options and their relatives will receive either 1) col-ACP 2) a supportive intervention (active control group) or 3) standard medical care (TAU). col-ACP comprises two steps: a) addressing various barriers of patients and relatives that discourage them from discussing end-of-life issues followed by b) a regular, structured ACP procedure. The col-ACP-intervention consists of 6 sessions. Primary endpoint is the patients’ quality of life 16 weeks after randomisation. Secondary endpoints include measurements of distress; depression; communication barriers; caregivers’ quality of life; existence of ACP or advance directives; the consistence of end of life care; and others. Patients will be followed up for 13 months. Multivariate analyses will be carried out. Qualitative evaluation of the intervention will be conducted.

**Discussion:**

Augmentation of a regular ACP program by a structured psycho-oncological intervention is an innovative approach to target barriers of communication about end-of-life issues. Study findings will help to understand the value of such a combined intervention in palliative care.

**Trial registration:**

ClinicalTrials.gov Identifier: NCT03387436 (Date of registration: 01/02/2018, retrospectively registered.

## Background

Cancer is one of the major causes of death in developed countries. According to the Global Cancer Observatory there were 18.1 million new diagnoses and 9.6 million cancer related death in 2018 [1]. A high quality of end-of-life care, according to individual patient’s wishes and values has become an important goal of palliative care [2–4]. To achieve these goals, patient’s preferences for end-of-life (EoL) care need to be known [5]. However, a considerable part of these patients cannot express wishes or values in their end-of-life period due to disease based cognitive impairment [6]. Therefore, communication about end-of-life care needs to be in time. Additionally, optimal communication between patients, their family and their physicians has been identified to be one of the most important aspects of medical care at the end of life [7, 8]. In fact, end-of-life discussions occur usually infrequent, too late and are often crisis-orientated and restricted to decisions about life-sustaining treatments, which can cause relevant distress in patients and relatives [6, 9]. Also, unmet needs during EoL care are commonly reported by bereaved family members [10]. Although advance directives were designed to diminish these problematic issues, they are less common than expected [11–13]. Additionally, traditional advance directives have shown several points of weakness in daily clinical routine [14].

To overcome these challenges, a great effort has been made since the 1990s establishing the idea of advance care planning (ACP). An international consensus supported by the European Association for Palliative care defines it as follows: “Advance care planning enables individuals to define goals and preferences for future medical treatment and care, to discuss these goals and preferences with family and health-care providers, and to record and review these preferences if appropriate” [15]. There is evidence that ACP-interventions can improve patient-related outcomes such as patient satisfaction with care, consistency of actual care and patients´ goals, quality of communication and shared decision-making [16].

However, implementation of ACP is facing many organisational and individual psychological barriers. The first include: lack of integration into workflow as well as lack of information and involvement of involvement of clinical carers in the ACP process and its documented outcomes. Additionally, system-wide implementation of complex ACP interventions remains difficult as a review of Lund et al. demonstrates [17]. Even if organisational barriers are low, ACP is not inquired as frequently as expected because of cultural and psychological barriers that hinder a frequent realization of ACP and communications about EoL wishes. Those barriers include fear of own death and of burdening relatives, unawareness of gravity of disease, “optimism”, aversive emotional responses to reflecting death and avoidance of end-of-life topics [7, 8, 18, 19].

A complex intervention might improve implementation of ACP and enable EoL care that is concordant with patient wishes. This complex ACP intervention should address barriers of engagement and can thus enhance EoL communication, ACP implementation and improve quality of life and EoL care in cancer patients with limited disease prognosis. A recent systematic review stated that multi-focal interventions may effectively remove barriers to end-of-life communication [16]. Such multi-focal interventions may include amongst others a) facilitation of communication by specifically trained personnel, b) advance care planning, c) structured communication roles, responsibilities and processes, d) summarising patient end-of-life preferences and values using a questionnaire with feedback of this summary document to clinicians and e) psychoeducation for patients and relatives [16, 19, 20].

Acknowledging these findings, the present randomised controlled trial investigates the effectiveness and efficacy of an innovative, collaborative ACP intervention regarding the quality of life of patients and relatives. The intervention targets psychosocial barriers of patients and relatives towards EoL discussions prior to a standardised ACP process (“beizeiten begleiten” [21] German adaptation of “respecting choices”). We hypothesise that combining psycho-oncological tools with standardised ACP can be an innovative approach to improve EoL care and quality of life of cancer patients, as this is a central goal of palliative care [22]. Because of its multi-professional aim the intervention is called **col**laborative **a**dvance **c**are **p**lanning (col-ACP).

The main research questions are:

- Can col-ACP improve quality of life in palliative patients and relatives?
- Does col-ACP influence distress, anxiety and communication barriers in patients and relatives and enhance consistent end-of-life care?
- Does col-ACP influence patient relative communication about EoL topics?

## Methods/design

### Study design and setting

The col-ACP-study is a randomised controlled trial designed to evaluate the efficacy and effectiveness of a collaborative advance care planning intervention. Three intervention arms will be compared longitudinally. We will screen and recruit palliative cancer patients from multiple settings of the German health care system. Our screenings sites are:

- University hospital of the Philipps-University Marburg – department of haematology/oncology, department of oncological urology, department of oncological gastroenterology, department of radiation therapy; patients are recruited from inpatient and outpatient settings
- Clinic for oncological rehabilitation “Sonnenblick”, Marburg
- Specialised ambulant palliative care team of the county Marburg-Biedenkopf

The respective sites were chosen, since – taken together - they cover approximately 90% of all specialised palliative care services for patients in our district (Marburg-Biedenkopf). Integrating all these sites in our recruitment strategy increases the representativeness of our approach.

### Study population

The study will recruit 270 adult cancer patients with non-curable disease. The trial is open to all neoplastic entities. Patients are screened with the `surprise question‘. Cancer patients with these characteristics have a high likelihood of dying in the next year [23]. However, patients are excluded if the treating physician is certain that the patient will not survive at least 4 months after randomisation. Exclusion criteria are the inability to perform the study intervention (e.g. ECOG ≥ 3), insufficient German language skills and cognitive inability to give informed consent.

### Study procedures

Medical treatment of patients is independent from the study procedures and will be carried out accordingly to the respective cancer guidelines. The study intervention is an addition to the medical treatment as usual.

**Fig. 1:**
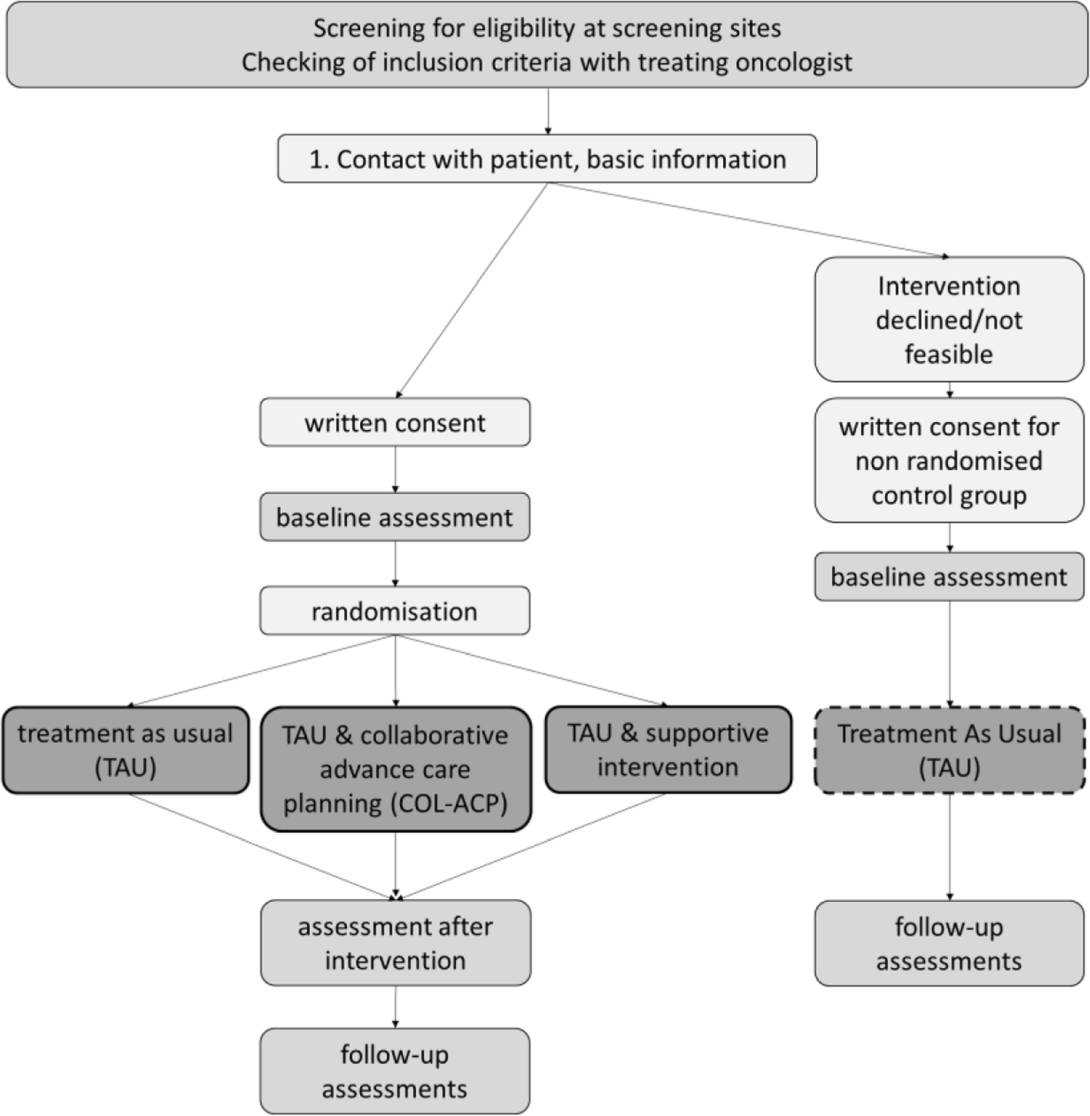
Study Design

Patients are screened for eligibility by their treating physician; this process is taking place individually at each screening site. Patients who meet inclusion criteria are approached by a licensed physician. They are informed in writing and verbally about the study and invited to partake with a relative or close friend. After written consent and collection of baseline assessment, patients are admitted to the study and randomised equally to one of three study arms (Fig. 1). Participation is voluntary and can be withdrawn at any time. All collected data can be requested and deleted by patients according to EU data privacy policy. Patients will be randomised in the following arms: Treatment as usual (TAU), active control group (ACG), study intervention (col-ACP). If patients decline to participate, they can contribute to the non-randomised control-group.

### Power calculation, sample size, recruitment

Sample size of 77 per group were calculated with a two-sided significance level of α = 5% to achieve a power of 80% to reject at least one comparison, when at least one difference between groups has an effect size of d = 0.5. This effect size was determined assuming a minimal important difference of 5 (71) and a standard deviation of approximately 10 (72). Assuming a drop-out rate (meaning no QoL (Quality of life) data available after intervention, see 7) of 20%, 90 patients per group should be recruited. We have to expect about 130 to 155 events of death in our patient population depending on the assumption of the one-year survival rate. The primary hypotheses will be analyzed using a linear mixed model, which will provide estimates for missing values. Compared to intention-to-treat analyses with last-observation-carried forward methods, this procedure provides better estimates for missing data using the full data sample (intention-to-treat sample), and addresses individual differences more adequately.

### Quality standards

#### Randomisation and blinding

Randomisation follows after completing baseline assessment. Randomisation is carried out by the department of biometry of the Philipps-University which is not engaged in the intervention or data collection. Stratification criterion is the participation of a relative. Research assistants helping to record the obtained data are blinded for the intervention groups.

#### Selection and attrition bias

In order to avoid a potential selection bias all palliative cancer patients at a screening site will be screened for eligibility. If pre-screened patients meet inclusion criteria they will be approached and asked to participate in the study. We examine potential attrition bias by carefully analysing drop-outs. In accordance with the intention-to-treat principle, the data of all patients randomised to the treatment groups will be analysed. When a drop-out occurs due to the death of a patient, bereaved relatives will be followed up if approved.

#### Control for allegiance

To ensure the comparability of the interventions, assessment occasions for col-ACP and active control group (ACG) will be identical. All therapists are clinical psychologists with advanced cognitive behavioural training and have comparable professional experience. ACP facilitation skills were trained in an 8-day course including medical, legal, ethical and psychological background of ACP by experienced trainers. Communication skills were trained with the help of trained actors. All therapists are certified by the German branch of the “Respecting Choices” program. Training includes a full treatment cycle (6 sessions) with at least one patient per treatment group using video feedback and professional supervision of each treatment session. During the entire study, therapists are under ongoing supervision by highly experienced psycho-oncologists and two ACP facilitation trainers. Both types of interventions are manualized and each therapist will treat a comparable number of patients in each group. Treatment fidelity is assessed and rated before approval of therapists to start in this trial. All sessions will be audiotaped and an amount of 20% will be selected randomly and rated by an independent rater to assess adherence and quality.

### Study arms

#### Treatment as usual

Patients included in this study arm receive standard care as usual in their health care setting and stage of disease. Patients are welcome to receive psycho-oncological support or ACP if offered by their usual health care providers. Study patients and relatives randomised to this arm will take part in assessment by questionnaire only.

### Interventions

Both types of intervention (col-ACP intervention and active control intervention) will be performed by psychologists with cognitive behavioural therapy training and additional training in ACP. Patients and relatives will have the same therapist throughout all sessions of the intervention. Session duration will be tailored to the patients’ physical abilities (30–90 min).

#### Col-ACP intervention

**Fig. 2:**
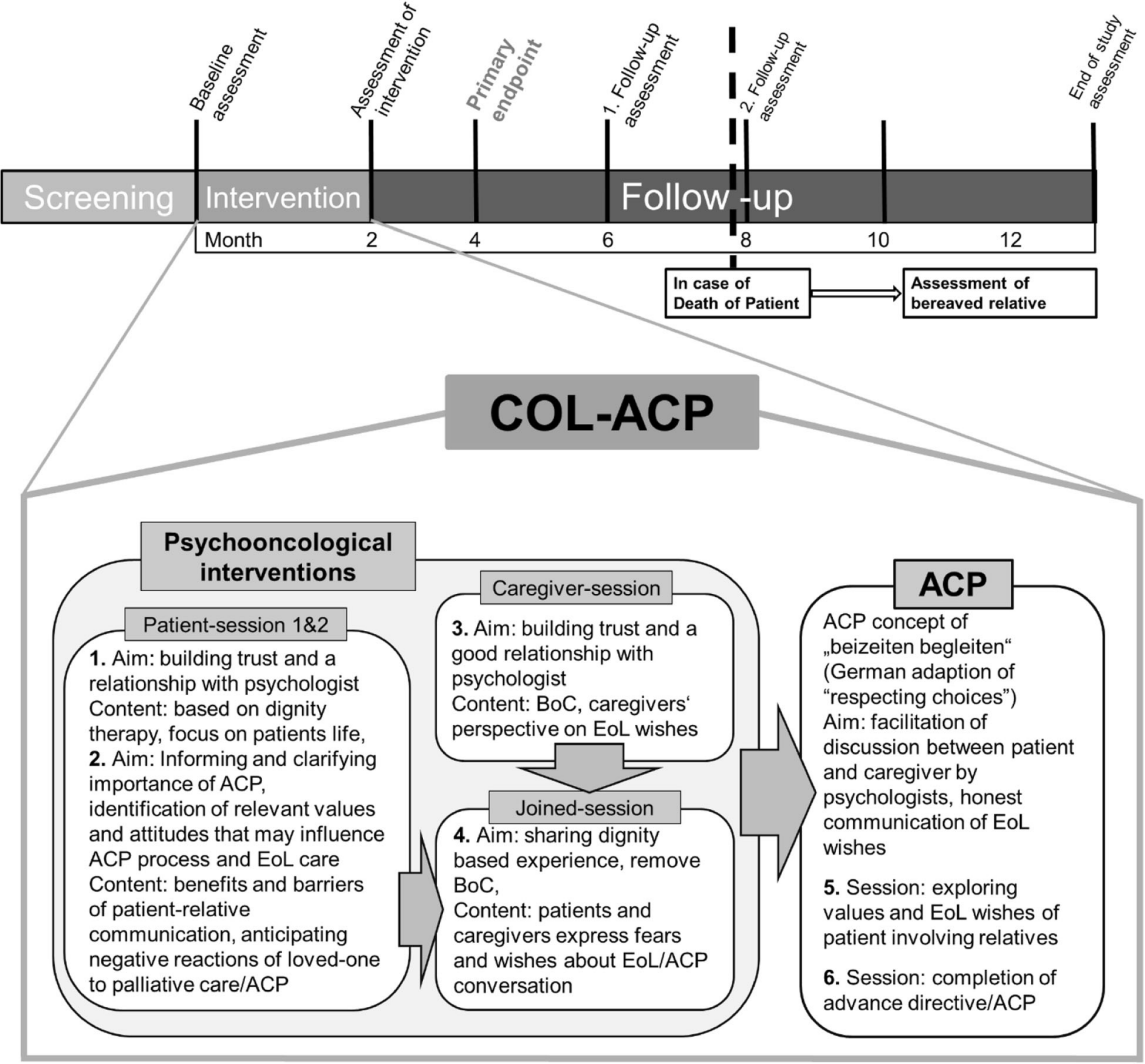
Design and content of the col-ACP intervention

The col-ACP intervention (see Fig. 2) is a combination of dignity-based interventions, cognitive behaviour-based interventions and a standardised advance care planning (ACP) procedure. The first two sessions for patients and one session for relatives are conducted in an individual setting. In the remaining sessions, patients and relatives should participate together.

- Session 1: The first session is oriented towards the Dignity Therapy question protocol (e.g. “Tell me a little bit about your life history; particularly the parts that you either remember most or think are the most important.” “Are there specific things that you would want your family to know about you?”) and will review important landmarks in the patient’s life [24, 25]. An individual graphic is created to illustrate and anchor patients´ answers and given to the patients.
- Session 2: In the second session, patients will be informed about the relevance of planning their end-of-life period. They will be interviewed concerning their need for and potential fears of EoL communication. Potential benefits, but also barriers for an efficient patient-relative communication and ACP are discussed (e.g. fears of “burdening” the other with EoL communication; anticipating negative reactions of the other to own preferred palliative care options). Identification and modification of these barriers will be based on intervention techniques of cognitive behavioural therapy, taking into account existential approaches.
- Session 3: One separate session with the same focus on communication barriers is conducted with the relative only, since incorporating the relatives’ perspective into the intervention is crucial.
- Session 4: The fourth session will include patients and relatives. It focuses on sharing the dignity-based experiences, and on jointly modifying barriers regarding to EoL communication. To achieve these goals, the most relevant patient issues will be discussed with the relative. Additionally, patients and relatives will be asked to express their respective fears and wishes for EoL communication during the session while the other is present. The clinical psychologist will moderate a discussion in order to accommodate both patients´ and relatives´ wishes and facilitate solutions in case of conflicting preferences. If patients or relatives voice concerns about EoL communication for fear of burdening the other, the psychologist will help to appraise the correctness of this assumption by eliciting feedback and will discuss short-term and long-term consequences of EoL discussions. Focusing on “family involvement” [26] and discussing relevant future goals to encourage a coping-focused perspective.
- Session 5 and 6: The fourth and fifth sessions focus on the ACP based standardised concept of “beizeiten begleiten” [21]. The concept is modified by referring to the obstacles of an efficient patient-relative communication that were identified in the previous sessions.

#### Active control intervention

The supportive intervention uses common factors of psychotherapy such as elicitation of affect, reflective listening, and feeling understood, but provides no explicit theoretical formulation to the patient. The psychologist tries to elicit and validate the patients’ affect for instance on the realization that there is no curative treatment option. However, topics are not specified, and patients can talk about anything that has affective valence to them. The sessions do not target the specific topics of the col-ACP intervention group (i.e. no special focus on ACP or end-of-life communication). Supportive interventions have been used as an unspecific control condition in several studies [27, 28]. All sessions are administered by the same personnel, relatives receive one session alone, the other five sessions are conducted with the patient. No sessions include both patients and relatives as these techniques of communication are specific for the col-ACP intervention.

### Measurements

Over the duration of 1 year, measurements are assessed in intervals of 8 or 10 weeks (see Table 1). After Baseline-assessment, patients will receive 7 follow-up questionnaires. The first follow up is conducted after the intervention and covers, among other endpoints, patient and relative satisfaction with the intervention. In order to be more flexible with the intervention the interval between baseline (T0) and first assessment (T1) is 10 weeks. The primary endpoint (questionnaire: T2) is collected 16 weeks after baseline assessment. From T2 on, patients will receive follow up (T3-T7) every 8 weeks until death or until 1 year after baseline assessment. Relatives willing to take part are assessed at Baseline, T1, T2 and after 1 year (T7). If the patient dies during follow up, relatives are asked to fill out a questionnaire assessing coping with grief and quality of death of the patient.

**Table 1:** Measurements of the study

	baseline		post-intervention		primary endpoint		follow-up	final assessment		assessment after death of patient
	patient	relative	patient	relative	patient	relative	patient only	patient	relative	relative only
Baselinedata	x	x								
FACT-G	x		x		x		x	x		
FACIT-PAL 14	x		x		x		x	x		
SF-12 v2	x	x	x	x	x	x	x	x	x	x
PHQ-9	x	x	x		x	x				
Peace-Scale	x		x		x		x	x		
Distress	x		x		x					
Barriers of communication	x	x	x	x						
Treatment Expectations	x	x			x	x				x
Evaluation of intervention^a^			x	x						
Existence of advance directive/(c)ACP	x				x			x		x
Conception of EoL care	x				x					
Other supportive interventions	x				x			x		x
CQOLC		x		x		x			x	x
QODD										x
Inventory of complex grief										x
Circumstances of death										x

^a^not applicable to TAU-arm

Measurement of endpoints is conducted by blinded research assistants at all measurement points. The questionnaires will be sent home; research assistants will support patients and relatives if needed. Baseline assessment also includes socio-demographic status, age, gender, tumour characteristics (ICD-Code), current treatment (chemotherapy, radiation therapy) and existence of living will. This information will be obtained by a standardised interview and from medical charts. Blinded research assistants entry the data and double check 20% of the data. Any protocol modifications will be communicated via ClinicalTrials.gov

### Primary endpoint

*Quality of Life* (QoL) of patients is chosen as primary endpoint, since improving or maintaining quality of life is the primary goal of palliative medicine [29]. Disease-specific QoL will be assessed 16 weeks after randomisation by the Functional Assessment of Cancer – General Version (FACT-G) [30]. The FACT-G covers four dimensions: physical well-being, social/family well-being, emotional well-being and functional well-being. It is frequently used in cancer clinical research as a primary endpoint [31] and has demonstrated its reliability and validity [32]. The FACT-G will be extended by items of the FACIT-Pal [33, 34]. This combination of items forms the FACIT-Pal 14 [33], a validated short form of the FACIT-Pal and well established in palliative care research [35, 36].

### Secondary endpoints

Secondary measures include the following assessments:

- *Generic quality of life* will be assessed with the Short Form-12 health survey (SF-12) [37]. The SF-12 allows computation of physical and mental health scores and has been used with cancer patients [38–40].
- *Depressive symptoms* will be measured with the Patient Health Questionnaire-9 (PHQ-9) [41]. This survey assesses severe symptoms of depression based on criteria by the DSM IV [42]. It consists of 9 items, a high sum score (range 0–27) indicates a high level of depression. A German version has been adapted and validated [43, 44].
- *Distress* will be assessed with the National Comprehensive Cancer Network Distress Thermometer (NCCN Distress Thermometer) [45]. It consists of a scale from 0 to 10, assessing overall psychosocial distress and was specifically developed for oncological patients.
- *Peace, Equanimity, and Acceptance in the Cancer Experience* will be measured with the Peace-Scale [46]. The Peace-Scale consists of two subscales, “Struggle with Illness subscale” and “Peaceful Acceptance subscale”. It has been developed to assess the emotional acceptance of a non-curable cancer disease.
- *Barriers of communication* will be assessed with a self-developed assessment tool. In literature reviews and a self-conducted qualitative study we identified several barriers of successful communication such as: fear of burdening loved-ones, inner willingness to talk about EoL issues, not finding the “right words”, timing, expectation of blindly understanding a relative’s wishes. A tool for assessing barriers of communication (BoC) was missing. As part of the project, we developed and validated the barriers of communication-scale.
- *Treatment expectation* will be assessed by a self-developed questionnaire, focussing on the patients´ and relatives’ expectation about the aim of treatment, meaning for example if patients expect to be cured by the treatment or if they expect it to improve, or worsen, their QoL.

#### End of life care

*Conception and wishes of/for end of life (care)* are assessed by a self-developed tool using multiple choice questions and open-ended questions. Patients are asked what kind of wishes they have for the end of their life, e.g. “Where do you want to die?” and “Who do you wish to be there when you die?”. Patients are in addition given the opportunity to make a list of wishes for their end of life care.

*Existence of advance directives or advance care planning* will be measured by a single self-developed item, which also asks patients from whom they got help with their AD (advance directives), or who facilitated ACP with them.

#### Relative-only variables

*Caregiver Quality of Life Index – Cancer Scale (CQOLC)* [47]*.* This special scale focusses on the impact of cancer on family caregivers. It has shown good psychometric properties in several settings including home hospice care [48].

#### Assessment after death of patient

*Quality of Dying and Death Questionnaire for Informal Caregivers (QODD-D)* [49, 50]. This scale is assessed after the death of a patient. It has several domains such as: Symptom Control, Preparation, Connectedness, and Transcendence and is validated in a German version [49].

*The Inventory of complicated Grief (ICG)* [51, 52] was developed to predict long-term functional impairments due to “complicated grief”. Its subscales measure grief, depression and background characteristics. The German version showed good psychometric properties [52].

*Concordance of EoL wishes and actual EoL care* is assessed in a structured interview with relatives and with a questionnaire which presents patients EoL wishes to relatives.

*Demographic information* such as age, gender, specific type of cancer and cancer treatment, formal education, etc. will be assessed in the clinical research form. Information will be obtained through patient interview and consultation of patient file.

### Qualitative evaluation

Both parts of the col-ACP study intervention contain very effective psycho-oncological techniques and practices, but combining them is a new concept. In order to evaluate the intervention properly, qualitative assessment is necessary. We will conduct semi-structured interviews with 10 to 15 patients and relatives in order to detect negative effects of col-ACP. The impact of col-ACP on patient-relative relationships and views about death and dying are also part of the interviews. To ensure unbiased review of the intervention, this research is carried out by an independent study team.

### Data analysis

For the primary endpoint, a linear mixed model will be fitted with independent variable intervention group and additional covariates recruitment setting, availability of a relative (strata from randomisation according to ICH E9), gender, ECOG and FACT-G at baseline. Analyses of the key secondary endpoint will be performed in the same way using linear mixed models. Additional analyses for the complete follow up data will be done using repeated measure models. An intention-to-treat population will be used for all analyses. Data analysis will be conducted by the department of biometry of the Ruhr-University which is not engaged in the intervention or data collection.

## Discussion

ACP can be a powerful tool to improve patient-centred care at the end of life, but system-wide implementation of ACP interventions proves to be difficult. This study presents a new approach towards ACP. It combines psycho-oncological interventions, which aim to improve patients´ and relatives´ communication about EoL care with standardised ACP facilitation. The col-ACP intervention is compared with an active control group and a “treatment as usual” group, all receiving standard cancer treatment. Primary outcome is the QoL of patients 4 months after randomisation. Secondary outcomes are among others: communication barriers, depressive symptoms in patients and relatives, concordance of care with patients´ wishes and quality of life of the relative.

We are aiming for a local and system-wide implementation of our intervention, recruiting patients in all settings of the German health care system. This includes inpatient and outpatient clinics, as well as a rehabilitation clinic and a specialised palliative care team. If the col-ACP intervention proves to be beneficial, it can be integrated in palliative care for cancer patients.

The study design of the col-ACP trial addresses sources of bias, such as selection and attrition bias, and we additionally control for allegiance. Selection bias are avoided by screening all palliative cancer patients at each screening site for inclusion criteria, all patients meeting inclusion criteria are then approached and asked for participation. Patients´ and relatives’consent is obtained before randomisation. While the facilitators that conduct the interventions (col-ACP and supportive intervention) cannot be blinded, patients are not told that the trial investigates an ACP-intervention. Patients in both intervention groups (col-ACP and supportive intervention) are told that the trial investigates the effects of “supportive conversations”. Study personnel involved in collection and management of data is blinded.

### Limitations

This study has limitations that need further consideration. First, our study collective is vulnerable towards loss of patients due to death before collection of the primary endpoint. Predicting patient’s life-expectancy in palliative cancer care is difficult, we therefore collaborate with experienced clinicians reviewing pre-screened patients. Second, in previous advance care planning trials quality of life as a primary endpoint has been difficult to improve [16]. QoL is influenced by many factors such as burden of disease, pain, and distress. We chose QoL as the primary outcome of our study because it is the primary and important goal of palliative care, but in addition investigate other highly relevant outcomes. Third, we conduct a complex intervention that needs highly skilled and trained facilitators. Our study personnel conducting the intervention are trained psychologists, which can be a challenge for broader implementation of our intervention.

## Conclusion

Nevertheless, combining psycho-oncological interventions with ACP might be a promising path to improve quality of life of palliative cancer patients and to address barriers of communication between patients and relatives. Our findings will provide implications for a broader implementation of collaborative advance care planning approaches in palliative cancer care.

## Data Availability

Data sharing is not applicable to this article as no datasets were generated or analysed during the current study.

## Abbreviations

ACG: Active control group
ACP: Advance care planning
AD: Advance directives
BoC: Barriers of communication
col-ACP: Collaborative advance care planning
CQOLC: Caregiver Quality of Life Index – Cancer Scale
ECOG: Eastern Co-operative Oncology Group Performance Status
EoL: End-of-life
FACIT-Pal: Functional Assessment of Chronic Illness Therapy Scale
FACT-G: Functional Assessment of Cancer – General Version
ICG: Inventory of complicated Grief
NCCN Distress Thermometer: National Comprehensive Cancer Network Distress Thermometer
Peace-Scale: Peace, Equanimity, and Acceptance in the Cancer Experience Scale
PHQ-9: Patient Health Questionnaire-9
SF-12: Short Form Health Questionnaire − 12
TAU: Standard medical care
QODD-D: Quality of Dying and Death Questionnaire for Informal Caregivers
QoL: Quality of life
